# A pan-cancer analysis of driver gene mutations, DNA methylation and gene expressions reveals that chromatin remodeling is a major mechanism inducing global changes in cancer epigenomes

**DOI:** 10.1186/s12920-018-0425-z

**Published:** 2018-11-06

**Authors:** Ahrim Youn, Kyung In Kim, Raul Rabadan, Benjamin Tycko, Yufeng Shen, Shuang Wang

**Affiliations:** 1 Department of Biostatistics, Mailman School of Public Health, Columbia University, New York, New York USA; 2 The Jackson Laboratory For Genomic Medicine, Farmington, Connecticut USA; 3 Department of System Biology, Columbia University, New York, New York USA; 4 Department of Biomedical Informatics, Columbia University, New York, New York USA; 5 Division of Genetics & Epigenetics, Hackensack University Medical Center, Hackensack, New Jersey USA; 6 Columbia Genome Center, Columbia University, New York, New York USA

**Keywords:** Pan-cancer analysis, TCGA, somatic mutation, DNA methylation, gene expression, methylation driver gene, expression driver gene, chromatic remodeling

## Abstract

**Background:**

Recent large-scale cancer sequencing studies have discovered many novel cancer driver genes (CDGs) in human cancers. Some studies also suggest that CDG mutations contribute to cancer-associated epigenomic and transcriptomic alterations across many cancer types. Here we aim to improve our understanding of the connections between CDG mutations and altered cancer cell epigenomes and transcriptomes on pan-cancer level and how these connections contribute to the known association between epigenome and transcriptome.

**Method:**

Using multi-omics data including somatic mutation, DNA methylation, and gene expression data of 20 cancer types from The Cancer Genome Atlas (TCGA) project, we conducted a pan-cancer analysis to identify CDGs, when mutated, have strong associations with genome-wide methylation or expression changes across cancer types, which we refer as methylation driver genes (MDGs) or expression driver genes (EDGs), respectively.

**Results:**

We identified 32 MDGs, among which, eight are known chromatin modification or remodeling genes. Many of the remaining 24 MDGs are connected to chromatin regulators through either regulating their transcription or physically interacting with them as potential co-factors. We identified 29 EDGs, 26 of which are also MDGs. Further investigation on target genes’ promoters methylation and expression alteration patterns of these 26 overlapping driver genes shows that hyper-methylation of target genes’ promoters are significantly associated with down-regulation of the same target genes and hypo-methylation of target genes’ promoters are significantly associated with up-regulation of the same target genes.

**Conclusion:**

This finding suggests a pivotal role for genetically driven changes in chromatin remodeling in shaping DNA methylation and gene expression patterns during tumor development.

**Electronic supplementary material:**

The online version of this article (10.1186/s12920-018-0425-z) contains supplementary material, which is available to authorized users.

## Background

Cancer arises through accumulation of somatically acquired genetic and epigenetic aberrations that lead to malignant transformation [1, 2]. Comprehensive characterization of somatic mutations in cancer genomes using next-generation sequencing technology has led to discoveries of cancer driver genes (CDGs) in human cancers [2]. The interplay between genetic and epigenetic alterations was only recently revealed through genome-wide scale genomic and epigenomic analyses. Specifically, genome-wide change of DNA methylation was observed in patients with mutations in epigenetic regulators [2–4], affecting both the global levels of 5-methyl-cytosine (5mC) and the precise DNA methylation patterns in diverse regulatory sequences across the genome [2, 3]. A recent study investigated associations between driver gene mutations and DNA methylation alterations across many cancer types [5], and identified associations between mutated driver genes and site-specific methylation changes as well as some genome-wide trends in specific cancer types. They further used these mutation-methylation associations to better define cancer subtypes. However, it remains largely unknown how the CDG mutations contribute to changes in cancer cell epigenomes on a pan-cancer level [6]. A better understanding of the connections between CDGs and altered cancer cell epigenomes is an important goal, particularly since mutations in epigenetic regulators could be novel targets for anti-cancer therapies [6].

Studies have integrated multi-scale omics data, including somatic mutation data, epigenomes, and transcriptomes across various cancer types to improve the mechanistic understanding of the interplay between cancer genome and cancer epigenome and transcriptome. An integrative analysis of DNA methylation data and gene expression data of various cancer types identified pan-cancer hypo- and hyper-methylated genes that are predictive of transcription as well as methylation-driven subgroups with clinical implications [7]. Another integrative analysis on a set of known epigenetic regulators with DNA methylation data and gene expression data from various cancer types identified key epigenetic regulators whose deregulation patterns are associated with genome-wide DNA methylation changes, which transcend cancer types [8].

Here we aim to improve our understanding of the connections between CDGs and altered cancer cell epigenomes and altered cancer cell transcriptome on pan-cancer level, and how these connections contribute to the known association between cancer epigenome and transcriptome. We used somatic mutation, DNA methylation, and gene expression data of 20 cancer types from The Cancer Genome Atlas (TCGA) project to identify CDGs that, when mutated, have strong associations with genome-wide methylation or expression changes across cancer types, which we refer as methylation driver genes (MDGs) or expression driver genes (EDGs). We identified 32 MDGs and found that most of them are either chromatin regulators (genes involved in chromatin remodeling) or ones that regulate the expression of or physically interact with chromatin regulators. We also identified 29 EDGs and found that 26 of them overlap with the 32 MDGs. We further investigated target genes’ methylation and expression alteration patterns that are associated with mutation status of these 26 overlapping driver genes and found that hyper-methylation of target genes’ promoters are significantly associated with down-regulation of the same target genes and hypo-methylation of target genes’ promoters are significantly associated with up-regulation of the same target genes. This finding shows that dysregulation of chromatin regulators is potentially an important mechanism that induces global change of DNA methylation and gene expression in tumor development.

## Methods

We downloaded somatic mutation data, DNA methylation 450K array data, and gene-level RNA-seq data of 20 tumor types with at least 100 samples available in all three data types from TCGA. For DNA methylation 450K array data, we conducted standard quality control steps removing CpG sites that overlap with known single nuclear polymorphisms (SNPs), sites on the sex chromosomes and sites with missing values for more than 5% of the tumor samples within a tumor type. After these steps, 370,877 CpG sites remained. We then corrected for the type I/II probe bias using the BMIQ algorithm [9]

### Selection of candidate CDGs

We obtained level 2 somatic mutation data of the above-mentioned 20 tumor types from Broad Institute TCGA Genome Data Analysis Center Firehose [10] and selected candidate CDGs using the MutSIG [11] algorithm that tests how frequently a gene is mutated in a tumor type comparing to the background mutation rate. We used the false discovery rate (FDR) < 0.1 to select candidate CDGs. We then assessed the functional impact of mutations at gene levels using the MutationAssessor [12] algorithm to further remove mutations classified as neutral. Additional steps were done for COAD and STAD when an abnormally large number of candidate CDGs remained (1,433 and 553, respectively) after these steps to avoid potential high false positive discovery rate of CDGs. Specifically, we only kept the genes that were identified in any of the other 18 tumor types as well as identified in the Cancer Gene Census [13] and the numbers of candidate CDGs in COAD and STAD then dropped to 193 and 67. The number of candidate CDGs selected in all 20 tumor types is provided in Additional file 1: Table S1.

To conduct pan-cancer analysis associating mutation and methylation/expression, within a tumor type, we selected CDGs that have mutations in at least 5 samples with matched methylation data or expression data in order to have not-too-sparse numbers in the mutated group. For matched mutation and methylation data, 445 CDGs were selected across the 20 tumor types. Here we analyzed somatic mutations at the gene level and a gene is considered mutated in a tumor sample as long as there is any mutation in this gene. Within these driver genes, the number of tumor types in which a driver gene was mutated in at least five samples varies from 1 to 15 (Additional file 2: Table S2) where most of the CDGs were mutated in only one or two tumor types. *TP53* was mutated in 15 tumor types and *PTEN* was mutated in 14 tumor types. For matched mutation data and expression data, 422 CDGs were similarly selected. Of them, 403 CDGs overlap with the CDGs selected for matched mutation data and methylation data. For the 422 CDGs, the number of tumor types in which a CDG is mutated in at least five samples varies from 1 to 14 (Additional file 2: Table S2), where *TP53* and *PTEN* were mutated in 14 tumor types.

### Pan-cancer analysis to identify MDGs

We described the details in the pan-cancer analysis associating driver genes and genome-wide methylation alterations across cancer types. Similar procedures with necessary modifications to associate driver genes and gene expression changes were described in the Additional file 3: Text S1.

### Associate CDGs and DNA methylation in one cancer type

For CDG *i*, let *A*_*i*_ denote the set of tumor types in which CDG *i* is mutated in at least 5 tumor samples with methylation data available. We then determine the hyper- or hypo-methylation status per CpG site by the mutation status of CDG *i* using the nonparametric Wilcoxon test. Since methylation levels range from 0 to 1 and are often bimodally distributed across tumor samples and the numbers of samples in the mutated and non-mutated groups are extremely unbalanced. With the Wilcoxon test, we define a set of genome-wide hyper-methylated sites *S*_*i,k*_+ whose methylation levels are significantly increased at significance level 0.01 in the mutated group comparing to the non-mutated group of CDG *i* in cancer type *k*. We similarly define a set of hypo-methylated sites *S*_*i,k*_−. Since the goal is not to identify specific CpG sites that are affected by the mutation status but to see how the mutation status is associated with genome-wide methylation changes, no multiple comparisons adjustment is applied to the site-level differential methylation association test.

To determine if mutation status of CDG *i* is significantly associated with genome-wide methylation changes in cancer type *k*, we calculate the *p*-value *pi*,*k*, which is the probability of observing the number of differentially (hyper- or hypo-) methylated sites ${n}_{i,k}^m=\left|\ {S}_{i,k}^{+}\cup {S}_{i,k}^{-}\right|$ or more that are associated with the mutation status of CDG *i* in cancer type *k* under the null hypothesis that the mutation status of CDG *i* is not associated with genome-wide methylation changes. To do so, we generate a “methylation null pool”, which has the number of differentially methylated sites under the null hypothesis. We first selected genes that were mutated in at least 5 samples with methylation data available within a tumor type. We then further selected only top 500 highly mutated genes within each tumor type for computational efficiency and also excluded the 445 CDGs selected above. We ended up with 7,019 mutation genes (those are considered as passenger mutation genes) across 20 tumor types in the “methylation null pool” (see Additional file 4: Table S3 for the number of mutation genes from each tumor type). The 7,019 mutation genes have similar mutation rate (average number of mutations in a cancer type) with that of the 445 CDGs. The average mutation rate of these 7,019 mutation genes is 0.082 with standard deviation (SD) 0.10 while the average mutation rate of the 445 CDGs is 0.085 with SD = 0.13 (*p*-value=0.54 from a t-test).

Within each cancer type, we calculated ${n}_{j_{null}}^m$, the number of differentially methylated sites that are associated with the mutation status of the methylation null gene *jnull*= 1,…,7019, which form the “methylation null pool”. The p-value *pi*,*k*, is then calculated as the proportion of numbers ${n}_{j_{null}}^m$ in the “methylation null pool” that is greater than or equal to the observed number of differentially methylated sites ${n}_{i,k}^m$, that is, *p*_*i,k*_= $\frac{1}{7019}\sum \limits_{j_{null}=1}^{7019}I\left({n}_{i,k}^m\le {n}_{j_{null}}^m\ \right)$, where *I*(.) is the indicator function.

To investigate the potential selection bias in the “methylation null pool”, we also generated the null distribution of number of genome-wide differentially methylated sites by randomly splitting tumor samples of a tumor type into mutation and non-mutation groups, varying the percentage of mutation from 5 to 40% based on the mutation rate of the TCGA 20 tumor types and calculated numbers of differentially methylated sites between the two groups. We repeated this 10 times for each percentage from 5 to 40%, increasing by 1%. Therefore, we ended up with 360*20 values for the number of differentially methylated sites across 20 tumor types. We found that these numbers are on average much smaller than those from the “methylation null pool” generated using passenger mutations, making the *p*-values of CDGs more significant. This indicates that there is some association between passenger mutations and global methylation changes that random sampling cannot capture. Therefore, the methylation null pool generated by using the passenger mutations rather than randomly splitting may represent a better null distribution. The MDGs identified this way are those associated with methylation changes beyond what is expected for passenger mutations.

We classify the effect of CDG *i* on genome-wide methylation in tumor type *k* as:

$$ \mathrm{CDG}\ i\ \mathrm{in}\ \mathrm{tumor}\ \mathrm{type}\ k=\left\{\begin{array}{l}\mathrm{genome}\hbox{-} \mathrm{wide}\ \mathrm{hyper}\hbox{-} \mathrm{methylated}\ \mathrm{if}\ {p}_{i,k}<0.05\&\left(|{S}_{i,k}^{+}|>|{S}_{i,k}^{-}|\right)\\ {}\mathrm{genoem}\hbox{-} \mathrm{wide}\ \mathrm{hypo}\hbox{-} \mathrm{methylated}\kern0.5em \mathrm{if}\ {p}_{i,k}<0.05\&\left(|{S}_{i,k}^{+}|\le |{S}_{i,k}^{-}|\right).\end{array}\right. $$

### Associate CDGs and DNA methylation across multiple cancer types

To calculate the p-value, *pi*, testing if CDG *i* is significantly associated with genome-wide methylation changes across multiple cancer types, we compare ${\sum}_{k\in {A}_i}{n}_{i,k}^m$, the observed total number of differentially methylated sites associated with CDG *i* summed over *Ai* cancer types, to B resampled values generated from the “methylation null pool” where we set B=one million. More specifically, for CDG *i* that was mutated in |*Ai*| number of tumor types, the null distribution is generated using the B sets of sum of |*Ai*| random samples from the “methylation null pool”. We then calculate *pi* as follows:

$$ {p}_i={\sum}_{b=1}^BI\left({\sum}_{k\in {A}_i}{n}_{i,k}^m\le {\sum}_{j=1}^{\mid {A}_i\mid }{n}_{r_{b,j}}^m\right)/B, $$

where *r*_*b*, *j*_ is a random number between 1 and 7,019 from the b^th^ resampling. We use Benjamini-Hochberg procedure to adjust for multiple comparisons on *p*_*i*_, which is done within groups of CDGs that were mutated in the same number of tumor types. The MDGs are then identified as those CDGs with adjusted *pi* < 0.05.

## Results

### TCGA 20 Cancer Types

We assembled somatic mutation data, HM450 DNA methylation data and gene-level RNA-Seq data (upper-quantile-normalized count data) of 20 tumor types with at least 100 samples available in all three data types from TCGA. This includes breast invasive carcinoma (BRCA), bladder urothelial carcinoma (BLCA), cervical squamous cell carcinoma (CESC), colon adenocarcinoma (COAD), glioblastoma (GBM), head and neck squamous cell carcinoma (HNSC), kidney renal clear cell carcinoma (KIRC), kidney renal papillary cell carcinoma (KIRP), acute myeloid leukemia (LAML), lower grade glioma (LGG), liver hepatocellular carcinoma (LIHC), lung adenocarcinoma (LUAD), lung squamous cell carcinoma (LUSC), pancreatic adenocarcinoma (PAAD), pheochromocytoma and paraganglioma (PCPG), prostate adenocarcinoma (PRAD), sarcoma (SARC), stomach adenocarcinoma (STAD), thyroid carcinoma (THCA), testicular germ cell tumor (TGCT), and uterine corpus endometrial carcinoma (UCEC)). For detailed steps on processing DNA methylation data and selecting candidate CDGs, see Methods. We refer candidate CDGs as CDGs from now on for notation simplicity.

### The Pan-Cancer Analysis

**Fig. 1:**
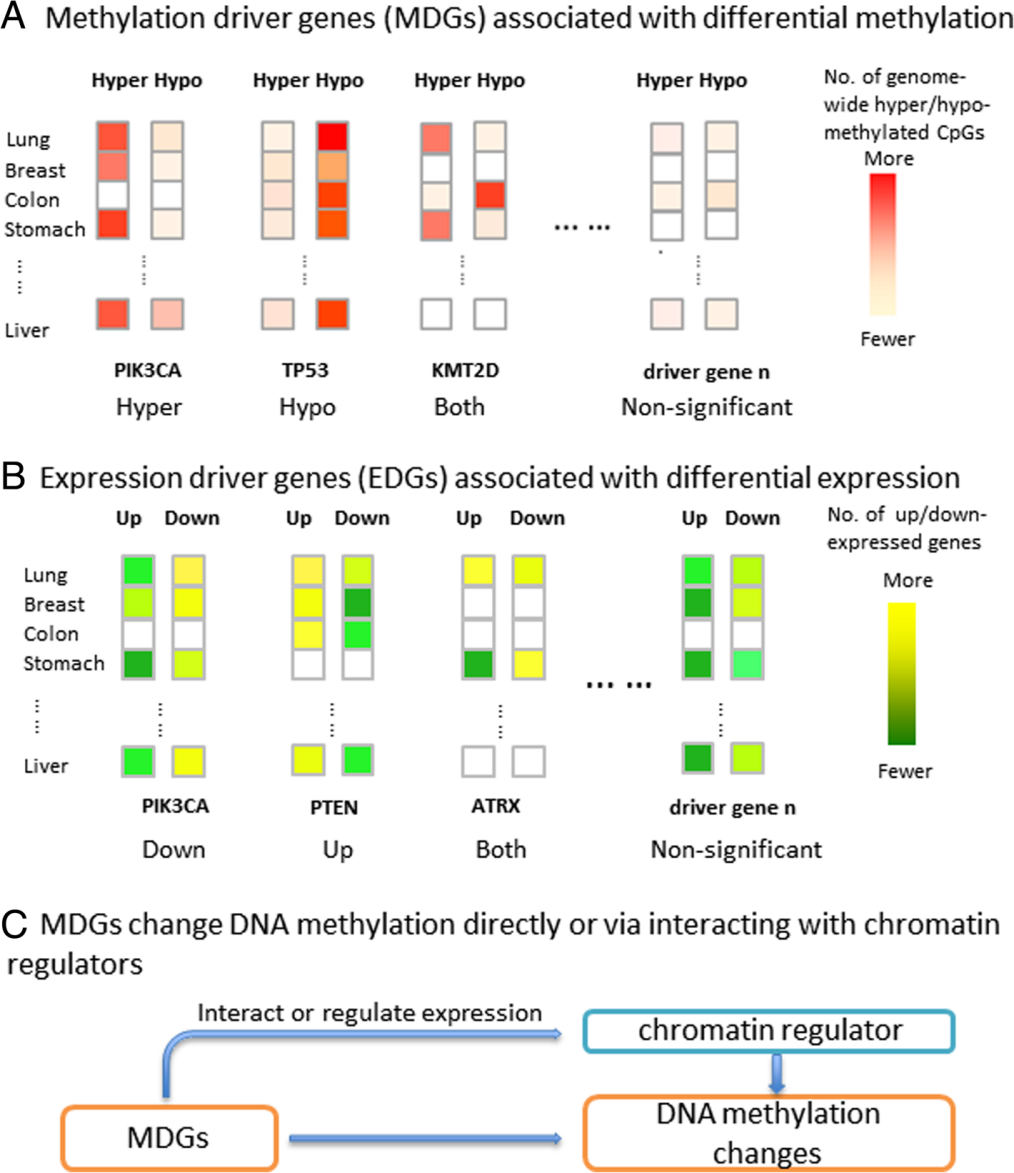
Rationale underlying the pan-cancer analysis to identify (**a**) MDGs that are associated with genome-wide methylation changes across cancer types and (**b**) EDGs that are associated with genome-wide expression changes across cancer types, with further analysis that reveals (**c**) MDGs mostly consist of chromatin regulators that directly affect the genome-wide methylation patterns or genes that regulate expression of or physically interact with chromatin regulator.

We conducted a pan-cancer analysis to identify methylation driver genes (MDGs)/expression driver genes (EDGs) that, when mutated, have strong associations with genome-wide methylation/expression changes across multiple cancer types through integrating somatic mutation and DNA methylation/gene expression data of 20 TCGA tumor types (Fig. 1a, b).

We then showed that some of the identified MDGs are chromatin regulators that directly affect the genome-wide methylation patterns and some are connected to chromatin regulators through either regulating their transcription or physically interacting with them as potential co-factors (Fig. 1c).

We first tested whether mutations in a CDG are significantly associated with changes in genome-wide methylation patterns in one cancer type. For this, we performed CpG-site-level association analysis within a cancer type, where a nonparametric Wilcoxon test was used since the numbers of samples in the mutated and non-mutated groups are extremely unbalanced and methylation measures were usually enriched at 0 and 1 [14]. We then used the number of genome-wide differentially methylated sites as the test statistic to measure degree of genome-wide methylation changes associated with the mutation status of a CDG for one cancer type. Note that we used significance level 0.01 to determine site-level association without multiple comparisons adjustment since the goal is to measure genome-wide degree of differential methylation due to mutation status but not to claim any associated CpG sites. To assess the significance of the genome-wide methylation changes by a CDG in one cancer type, we first generated an empirical null distribution with numbers of genome-wide differentially methylated sites by mutations of non-CDGs and then calculated the p-value *pi*,*k* for CDG *i* in cancer type *k* by comparing the number of genome-wide differentially methylated sites by the mutation of CDG *i* in cancer type *k* with the empirical null distribution*.* We then classify the effect of CDG *i* in tumor type *k* as hyper-methylated if *pi*,*k*<0.05 and the number of genome-wide hyper-methylated sites is greater than that of hypo-methylated sites or hypo-methylated if *pi*,*k*<0.05 and the number of genome-wide hypo-methylated sites is greater than that of hyper-methylated sites. Finally, to determine the significance of genome-wide methylation changes across multiple cancer types by a CDG, we compare the observed total number of differentially methylated sites associated with a CDG summed over all cancer types with its null distribution to calculate the p-value *pi*. We use Benjamini-Hochberg procedure to adjust for multiple comparisons for *pi*, where the adjustment is done within the group of CDGs that were mutated in the same number of cancer types. The MDGs are then identified as those CDGs with adjusted *p*-values < 0.05. Similar steps are applied to mutation and expression data to identify EDGs. Detailed steps of how to identify MDGs/EDGs are provided in the Methods.

### Thirty-two MDGs were identified that, when mutated, have strong association with genome-wide methylation changes across 20 cancers

The pan-cancer analysis of the 20 TCGA cancer types identified 32 MDGs (Table 1). For the complete list of CDGs whose mutation states were significantly associated with genome-wide methylation changes within each cancer type (gene *i* with *pi*,*k*<0.05 in the cancer type *k*), see Additional file 5: Table S4. The genes in Table 1 and Additional file 5: Table S4 highly overlap with the genes identified as the CDGs whose mutation states are associated with genome-wide methylation changes by Chen et al. [5]. They used Principal Component Analysis (PCA) to identify driver genes whose mutations are associated with the top five PCs within each cancer. Although the two methods used different approaches, the identified genes are very similar, providing further validation of the results.

**Table 1:** The identified 32 MDGs

MDGs	|A_i_|	|T_i_|	P_i_	D_i_	T^-^_i_	T^+^_i_
*TP53*	15	8	< e-06	both^a^	BLCA BRCA HNSC LIHC LUAD STAD UCEC	LGG^a^
*PTEN*	14	3	0.00147	both	LGG	STAD UCEC
*RB1*	11	2	0.00861	both	LGG	BLCA
*PIK3CA*	11	1	0.0277	hyper		STAD
*ARID1A*	10	1	0.0401	hyper		STAD
*KRAS*	8	1	1.2e-05	hypo	TGCT	
*KMT2D*	8	2	0.00575	both	BLCA	STAD
*NF1*	6	2	0.000227	hypo	LGG PCPG	
*CTNNB1*	6	2	0.00185	hypo	LIHC UCEC	
*SETD2*	5	2	0.00351	hyper		KIRC KIRP
*KMT2C* ^b^	5	1	0.00681	hyper		STAD
*EGFR*	4	1	9.5e-05	hypo	LGG	
*HRAS*	4	2	0.000266	both	PCPG	HNSC
*BRAF*	4	2	0.00474	both	THCA	COAD
*IDH1*	3	2	< e-06	hyper		GBM LGG
*CIC*	3	1	1.3e-05	hyper		LGG
*NRAS*	3	2	1.4e-05	both	TGCT	THCA
*RNF43*	3	2	5.1e-05	both	STAD	COAD
*ATRX*	3	1	0.000487	hyper^a^		LGG^a^
*ZBTB20*	3	2	0.00151	hyper		COAD STAD
*NOTCH1*	3	1	0.00189	hyper		LGG
*CDH1*	3	2	0.00213	hyper		BRCA STAD
*KEAP1*	3	1	0.00578	hypo	LUAD	
*SMARCA4*	3	1	0.00687	hypo	LUAD	
*FOXA1* ^b^	3	1	0.00953	hypo	PRAD	
*EPHA2* ^b^	3	1	0.0104	hyper		STAD
*KIT*	2	1	< e-06	hypo	TGCT	
*KMT2B*	2	2	0.000511	both	STAD	COAD
*FGFR3* ^b^	2	1	0.000697	hypo	BLCA	
*STK11* ^b^	2	1	0.00193	hypo	LUAD	
*NSD1*	1	1	< e-06	hypo	HNSC	
*BAP1* ^b^	1	1	0.000142	hyper		LIHC

|*Ai*|: number of tumor types in which CDG *i* is mutated in ≥ 5 samples with available methylation data;

|*Ti*|: number of tumor types whose genome-wide methylation levels are significantly associated with the mutation status of CDG *i*;

*p*_*i*_: *p*-value testing if CDG *i* is significantly associated with genome-wide methylation changes across tumor types;

*Di*: direction of methylation changes associated with mutation status of CDG *i*;

T^+^_i_: tumor types that are hyper-methylated by CDG *i*;

T^-^_i_: tumor types that are hypo-methylated by CDG *i*;

^a^: Further stratified analysis by *IDH1* mutation status in LGG tumor samples suggests an opposite direction from hyper- to hypo-methylation;

^b^: genes that are not overlapping driver genes

__ : genes that are identified as associated with genome-wide patterns of aberrant methylation by Chen et al. [5]

**Fig. 2:**
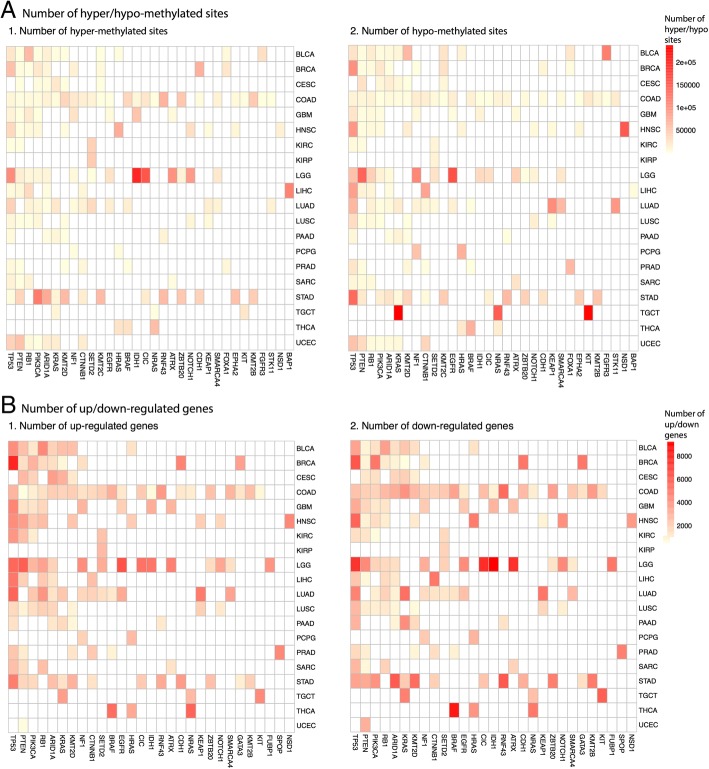
**a** Number of genome-wide hyper- (1) and hypo-methylated (2) sites that are associated with the mutation status of the 32 identified MDGs (columns) for each of the 20 TCGA tumor types (rows). (**b**) Number of genome-wide up-regulated- (1) and down-regulated- (2) genes that are associated with the mutation status of the 29 identified EDGs (columns) for each of the 20 TCGA tumor types (rows). The color code represents the number of differentially methylated sites/differentially expressed genes. Only driver genes that were mutated in ≥ 5 samples for the given tumor type were colored

The 32 MDGs were mutated with different frequencies in each cancer types (Additional file 6: Figure S1) and the mutation status of the 32 MDGs is associated with different genome-wide number of hyper- and hypo-methylated sites (Fig. 2a). Cancer types COAD and STAD have the highest mutation rate with many of the identified MDGs being mutated. KIRP, PCPG, TGCT and THCA have the smallest number of mutated MDGs. In CESC, LUSC, PAAD, and SARC tumor types, genome-wide methylation patterns were not significantly affected by mutations of any of the identified 32 MDGs, potentially due to small sample sizes or fewer number of CDGs. *TP53* mutations are associated with significant genome-wide methylation changes in 8 out of the 15 tumor types in which it was mutated in more than 5 samples (Table 1). Among these 8 tumor types, more CpG sites were hypo-methylated in all but LGG. Instead, in LGG, *TP53* mutations are associated with more hyper-methylated CpG sites. However, almost all LGG tumors with *TP53* mutations also have *IDH1* mutations (Additional file 6: Figure S1), which are known to lead to hyper-methylation in LGG [15–17]. *IDH1* is also identified as one of the 32 MDGs, where in GBM and LGG, it is associated with more CpGs to be hyper-methylated. Given the prominent role of *IDH1* in LGG, we stratified LGG tumor samples by the *IDH1* mutation status and further examined the effect of the other 31 MDGs within the *IDH1* mutation stratum and the *IDH1* wild-type stratum and found that *TP53* mutations are now significantly associated with more hypo-methylation genome-wide in each stratum (Additional file 3: Text S1). Similar stratified analyses were conducted in all other tumor types whose genome-wide methylation patterns were significantly associated with mutations of the identified MDGs. Similar patterns as in the non-stratified analysis were observed (Additional file 7: Table S5).

### Many MDGs are known chromatin regulators or the ones that regulate the expression of or physically interact with chromatin regulators

Among the identified 32 MDGs, 8 are known chromatin regulators that are either histone modification enzymes (*KMT2D*, *KMT2C*, *KMT2B*, *NSD1*, and *SETD2*), or part of ATP-dependent chromatin remodeling complexes (*ARID1A*, *ATRX*, and *SMARCA4*, all from the SWI/SNF family [18]).

We hypothesize that among the remaining 24 MDGs, some are “epigenetic modulators” in that these genes change genome-wide methylation patterns by regulating the expression of chromatin regulators/DNA methyltransferases, or through physically interacting with these epigenomic regulators as cofactors. To test this hypothesis, we examined whether mutations of these 24 MDGs are associated with the expression changes of known epigenomic regulator genes across the 20 tumor types, where we used the exon level RNA-Seq data of the 20 tumor tissue types from TCGA. We also investigated if epigenomic regulator genes are over-represented among genes that physically interact with these 24 MDGs.

We created two lists of known chromatin regulator genes. List A has 720 DNA/RNA, histone and chromatin-modifying enzymes and their co-factors from the EpiFactors database [19]. List B has 18 epigenetic regulators that were identified as the master regulators of global DNA methylation by Yang *et al.* [8], including *EYA4*, *SETBP1*, *PRDM2*, *PRDM5*, *CBX7*, *DUSP1*, *KAT2B*, *RAD54L*, *WHSC1*, *EZH2*, *UHRF1*, *PCNA*, *TTF2*, *KDM1A*, *SUV39H2*, *HDAC1*, *TDG* and *TET3*, plus the DNA methyltransferase *DNMT1*, *3A*, *3B* that were not identified as master regulators by Yang *et al.* [8].

**Fig. 3:**
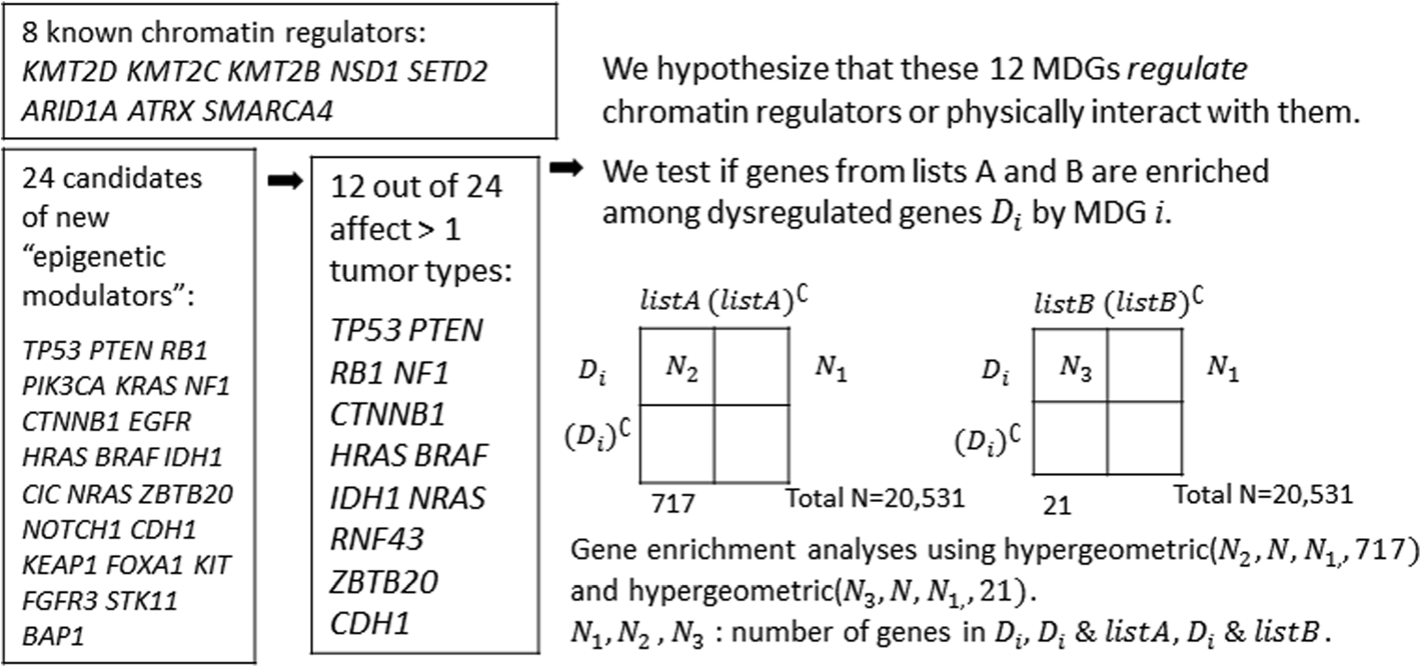
Steps to test if chromatin regulators are enriched among the dysregulated target genes associated with the mutation status of the identified MDGs

Among the remaining 24 MDGs that are potentially new “epigenetic modulators”, 12 are associated with genome-wide methylation changes in more than one cancer type (Table 1), including *TP53*, *PTEN*, *RB1*, *NF1*, *CTNNB1*, *HRAS*, *BRAF*, *IDH1*, *NRAS*, *RNF43*,*ZBTB20*, and *CDH1*. To focus on pan-cancer effects, we worked on only these 12 driver genes that are associated with genome-wide methylation changes in more than one cancer types. For each of these 12 genes, we first identified genome-wide target genes whose expression levels were dysregulated by the mutation status commonly across tumor types. We compared the expression levels of all genes between mutated and non-mutated groups of a MDG using a two-sample t-test and identified target genes that show significantly differential expressions (*p*-value <0.05) in all tumor types whose methylation patterns are associated with the mutation status. Similarly, since the goal here is not to identify specific target genes that are affected by the MDGs but to quantify degree of dysregulation by MDGs through number of target genes that are commonly dysregulated across cancer types, we used a loose p-value cutoff without considering multiple comparisons. We then examined if known chromatin regulators in lists A and B were over represented among the genome-wide dysregulated target genes by a MDG. The expression data were only available for 717 out of the 720 genes in list A, but were available for all 21 genes in list B. We used a hypergeometric distribution to calculate p-values for the enrichment of known chromatin regulators and described the procedure to examine this mechanistic hypothesis in more details in Fig. 3.

We found that among the genome-wide dysregulated target genes by each of the 12 MDGs, known chromatin regulators were clearly enriched (Table 2). In addition, 7 out of the 12 MDGs are associated with differential expression of the *DNMT* genes. Additionally, the results in Table 2 confirm some previously known interactions between MDGs and chromatin regulators. For example, *TP53* mutations are associated with upregulated *KDM1A* expression levels across all tumor types whose genome-wide methylation patterns are also significantly associated with *TP53* mutations. *KDM1A* is known to physically interact with *TP53* [20] and it demethylates histone lysine residues 9 of histone 3, which in turn leads to extensive hypo-methylation in that region [21]. This analysis suggests that *KDM1A* may play a role in the association between *TP53* mutations and genome-wide hypo-methylation changes across tumor types. Other notable associations that were confirmed by results in Table 2 include interactions between *RB1* and *DNMT1*, and between *RAS* genes (*HRAS*, *NRAS*) and *HDAC1* [22].

**Table 2:** MDGs dysregulate expression levels of chromatin regulators

MDGs	|T_i_|	N_1_ (# of dysregulated genes)	N_2_ (# of dysregulated genes in list A)	Enrichment Pvalue_A_	N_3_ (# of dysregulated genes in list B)	Enrichment Pvalue_B_	Genes in list B that are dysregulated^a^	Genes in list B that are differentially methylated^b^
*TP53*	8	233	19	0.00056	5	3.2e-06	*CBX7 RAD54L TTF2 KDM1A SUV39H2*	
*PTEN*	3	1,534	81	0.00012	9	9.1e-06	*DNMT1 DNMT3A SETBP1 PRDM5 CBX7 RAD54L EZH2 PCNA HDAC1*	*DNMT3A PRDM5 HDAC1*
*RB1*	2	1,447	83	5.2e-06	4	0.056	*DNMT1 EYA4 EZH2 PCNA*	*PCNA*
*NF1*	2	766	36	0.044	0	1		
*CTNNB1*	2	5,515	207	0.12	12	0.0033	*DNMT1 SETBP1 PRDM2 PRDM5 CBX7 KAT2B RAD54L WHSC1 EZH2 UHRF1 TDG TET3*	*PRDM2 CBX7*
*HRAS*	2	1,137	63	2.0e-04	3	0.11	*PRDM2 RAD54L HDAC1*	*PRDM2*
*BRAF*	2	3,128	133	0.008	8	0.0091	*DNMT1 DNMT3A DNMT3B KAT2B WHSC1 EZH2 UHRF1 KDM1A*	*DNMT3A UHRF1*
*IDH1*	2	3,560	208	2.8e-15	2	0.9	*SUV39H2 TET3*	
*NRAS*	2	1,609	87	2.8e-05	1	0.82	*HDAC1*	
*RNF43*	2	3,212	157	4.4e-06	8	0.011	*DNMT3A DNMT3B PRDM2 RAD54L WHSC1 UHRF1 HDAC1 TET3*	*DNMT3A DNMT3B PRDM2 WHSC1*
*ZBTB20*	2	1,563	78	0.00088	6	0.0039	*DNMT3A RAD54L WHSC1 UHRF1 TTF2 HDAC1*	*DNMT3A WHSC1 UHRF11*
*CDH1*	2	2,792	136	2.7e-05	14	3.3e-08	*DNMT3B SETBP1 PRDM2 CBX7 DUSP1 RAD54L WHSC1 EZH2 PCNA KDM1A SUV39H2 HDAC1 TDG TET3*	*PRDM2 CBX7 WHSC1 KDM1A*

|*Ti*|: number of tumor types whose genome-wide methylation levels are significantly associated with the mutation status of CDG *i*;

*N*_*1*_: number of genes whose expression levels are dysregulated by MDG *i* in all |*Ti*| tumor types;

*N*_*2*_: number of genes in list A that are dysregulated in all |*Ti*| tumor types;

*N*_*3*_: number of genes in list B that are dysregulated in all |*Ti*| tumor types;

Enrichment Pvalue_A_, and Pvalue_B_ are calculated using hypergeometric distributions testing if genes in lists A and B are enriched among genome-wide differentially expressed target genes;

^a^Genes in list B that are dysregulated in all |*Ti*| tumor types;

^b^Genes in list B that are differentially methylated in all |*Ti*| tumor types.

We next investigated if the epigenomic regulator genes in lists A and B are over represented among genes that physically interact with these 12 MDGs. To do so, we first obtained a list of genes that physically interact with each of them from the HumanMine database [23]. We then tested if the epigenomic regulator genes in lists A and B are over-represented among them. Since the number of physically interacting genes was too small for some MDGs, the enrichment analysis was only conducted for the genes in list A that had enough overlap with the interacting genes. We found that known epigenomic regulator genes in list A were highly enriched in the lists of interacting genes of the 12 MDGs (Table 3). These results support our hypothesis that these 12 MDGs that are not known chromatin regulators but are associated with changes in epigenomes either through regulating expression of epigenomic regulators or through physically interacting with them.

**Table 3:** Chromatin regulators are enriched in genes that physically interact with MDGs

MDGs	|T_i_|	N_4_ (# of physically interacting genes)	N_5_ (# of physically interacting genes in list A)	Enrichment Pvalue_A_^i^
*TP53*	8	923	192	0
*PTEN*	3	224	19	0.00035
*RB1*	2	250	64	0
*NF1*	2	29	3	0.079
*CTNNB1*	2	364	63	0
*HRAS*	2	86	6	0.08
*BRAF*	2	54	8	0.00053
*IDH1*	2	49	7	0.0015
*NRAS*	2	36	3	0.13
*RNF43*	2	18	7	1.4e-06
*ZBTB20*	2	17	1	0.45
*CDH1*	2	144	18	2.9e-06

|*Ti*|: number of tumor types whose genome-wide methylation levels are significantly associated with the mutation status of CDG *i*;

*N*_*4*_: number of genes physically interact with MDG *i*;

*N*_*5*_: number of genes physically interact with MDG *i* that are also in list A;

Enrichment Pvalue_A_^i^ is calculated using a hypergeometric distribution testing if genes in list A are enriched among selected physically interacting genes.

We also investigated whether differential expression of target genes in list B of 21 epigenetic regulators are directly or indirectly associated with differential methylation of the same genes. We found only a small fraction of genes in list B whose expression and methylation levels are both associated with the mutation status of the MDGs (Table 2), which suggests that the differential expression of these target genes may be directly associated with mutations of these MDGs instead of being indirectly associated through changes in their methylation patterns. We further investigated mutation status of genes in list B to examine if the mutations affect their expression or methylation levels directly and found that the majority of genes in list B were rarely mutated across tumor types (Additional file 8: Table S6).

Although *CIC* was not included in the above analyses since it was mutated only in LGG, due to its important role in LGG tumors, we examined how *CIC* regulates expressions of target genes and found that chromatin remodeling genes in list A were significantly enriched among dysregulated target genes, in both full LGG tumor samples and in stratified samples by *IDH1* mutation status (Additional file 9: Table S7).

### Twenty-nine EDGs were identified, out of which, 26 overlaps with the identified 32 MDGs

We conducted similar pan-cancer analysis to associate driver genes and gene expression across the 20 TCGA cancer types. We identified 29 CDGs as the expression driver genes (EDGs) that, when mutated, are significantly associated with genome-wide expression changes across multiple cancer types (Table 4). The mutation status of these 29 EDGs is associated with different genome-wide number of up- and down-regulated genes (Fig. 2b). For the complete list of CDGs whose mutation states were significantly associated with genome-wide expression changes within each cancer type, see Additional file 10: Table S8.

**Table 4:** Identified 29 EDGs

EDGs	∣*A*′_*i*_∣	∣*E*_*i*_∣	*p*′_*i*_	*B*_*i*_	${E}_i^{-}$	${E}_i^{+}$
*TP53*	14	11	< e-06	both	HNSC LGG SARC	BLCA BRCA COAD GBM KIRC LIHC LUAD STAD
*PTEN*	14	1	0.00299	up		LGG
*PIK3CA*	11	2	0.00536	down	BRCA STAD	
*RB1*	10	3	9e-05	up		BLCA LGG LUAD
*ARID1A*	10	2	0.00557	both	STAD	CESC
*KRAS*	8	4	4e-06	down	COAD LUAD PAAD TGCT	
*KMT2D*	8	1	0.0265	down	STAD	
*NF1*	6	1	0.00274	up		LGG
*CTNNB1*	5	1	0.00572	down	LIHC	
*†SETD2*	5	0	0.00833	NA	NA	NA
*BRAF*	4	2	1.1e-05	down	COAD THCA	
*EGFR*	4	2	5.3e-05	up		LGG LUAD
*HRAS*	4	3	7e-05	down	HNSC PCPG THCA	
*CIC*	3	1	< e-06	down	LGG	
*IDH1*	3	2	< e-06	both	LGG	GBM
*RNF43*	3	2	1e-06	down	COAD STAD	
*ATRX*	3	2	2e-06	both	LGG	GBM
*CDH1*	3	2	7e-06	both	BRCA	STAD
*NRAS*	3	2	0.000199	up		TGCT THCA
*KEAP1*	3	1	0.000809	down	LUAD	
*ZBTB20*	3	2	0.00119	down	COAD STAD	
*NOTCH1*	3	2	0.00182	down	HNSC LGG	
*SMARCA4*	3	1	0.0108	up		LUAD
*GATA3* ^a^	3	1	0.0138	down	BRCA	
*KMT2B*	2	2	0.000317	down	COAD STAD	
*KIT*	2	1	0.000889	down	TGCT	
*FUBP1* ^a^	1	1	< e-06	down	LGG	
*SPOP* ^a^	1	1	< e-06	down	PRAD	
*NSD1*	1	1	0.000432	up		HNSC

∣*A*′_*i*_∣ = number of tumor types in which EDG *i* is mutated in ≥ 5 samples with expression data;

∣*E*_*i*_∣ = number of tumor types whose genome-wide expression levels are significantly associated with CDG *i*;

${p}_i^{\prime }=$ p-value testing if CDG *i* is significantly associated with genome-wide expression changes across tumor types;

*Bi* is the direction of change of expression levels associated with the mutation status of CDG *i*;

${E}_i^{+}=$ tumor types that are up-regulated by CDG *i*, ${E}_i^{-}$= tumor types that are down-regulated by CDG *i*;

^a^ : genes that are not overlapping driver genes.

†Note that *SETD2* gene has a significant p-value ${p}_i^{\prime }$ for testing association of genome-wide expression changes across multiple tumor types, but there is not a specific tumor type in which *SETD2* mutation is significantly associated with genome-wide expression changes.

Of the 29 EDGs, 26 overlap with the 32 MDGs. To understand this high rate of overlap, within each cancer type, we examined the overlap between CDGs that are significantly associated with genome-wide methylation changes and CDGs that are significantly associated with genome-wide expression changes, and found they overlap highly. Moreover, there is a high correlation between the number of differentially methylated sites and the number of differentially expressed genes by each CDG (Additional file 11: Table S9), which implies a close connection between genome-wide methylation changes and genome-wide expression changes.

We further investigated patterns of target genes’ methylation in promoter regions and target genes’ expression changes of the 26 overlapping driver genes. A target gene is hyper-methylated if the number of hyper-methylated sites is larger than that of hypo-methylated in the promoter region of the gene (1,500 base pairs upstream of the transcription start site) and hypo-methylated otherwise. If there are the same numbers of hyper-/hypo-methylated sites or no hyper/hypo-methylated sites in the promoter region, the gene is considered not differentially methylated. The signature patterns of target genes’ methylation and expression changes by the overlapping driver genes could be hyper-methylated and up-regulated, the “++” pattern; hyper-methylated and down-regulated, the “+-” pattern; hypo-methylated and up-regulated, the “-+” pattern; and hypo-methylated and down- regulated, the “--” pattern. We used a hypergeometric distribution to calculate p-values for the enrichment of each pattern in a cancer type and combined per tumor type p-values using the Fisher’s method (Additional file 12: Figure S2, Table 5).

**Table 5:** Patterns of target genes’ promoter regions methylation and expression changes by mutations of the overlapping driver genes across tumor types

Overlapping driver genes	∣*T*_*i*_∣	∣*E*_*i*_∣	∣*T*_*i*_ ∩ *E*_*i*_∣	∣*DM*∣	∣*DE*∣	$\frac{\mid DM\cap DE\mid }{\mid DE\mid }$ (%)	*p*(*DM* ∙ *DE*)	*p*(−−)	*p*(+−)	*p*(−+)	*p*(++)	p.methyl	p.exp
*TP53*	8	11	7	10389	10066	52	2.8e-32	1	9.7e-95	7.1e-65	1	0	0
*PTEN*	3	1	1	12259	10293	61	0.014	1	1e-15	1e-15	1	2.98e-10	0
*RB1*	2	3	1	9049	8062	46	0.0066	0.81	0.05	1e-15	1	0	0
*PIK3CA*	1	2	1	13933	5822	73	5e-12	1	1e-15	5.4e-05	1	1.93e-11	0
*ARID1A*	1	2	1	9487	8573	53	1e-15	0.98	1e-15	0.29	1	0	0
*KRAS*	1	4	1	15041	8427	73	1	1	7.5e-12	1	0.87	0	0
*KMT2D*	2	1	1	9802	8485	52	4.3e-14	1	1e-15	0.014	1	7.37e-10	0
*NF1*	2	1	1	10288	7435	53	9e-07	1	1e-15	1e-15	1	0	0
*CTNNB1*	2	1	1	7583	7591	37	0.64	1	1e-15	1	1	0	0
*EGFR*	1	2	1	13156	10606	66	0.0096	1	1e-15	1e-15	1	7.63e-07	0
*HRAS*	2	3	2	8758	6300	47	3.7e-19	0.97	7e-29	8.1e-20	1	0	0
*BRAF*	2	2	2	9120	10092	47	1.3e-06	0.96	1.3e-14	7e-29	1	0	9.99e-16
*IDH1*	2	2	2	10596	10134	55	6.6e-16	1	7e-29	7e-29	1	0	0
*CIC*	1	1	1	14066	12718	71	1.2e-08	1	1e-15	1e-15	1	0	0
*NRAS*	2	2	1	8413	10581	43	5.6e-06	1	1e-15	1e-15	1	0	0
*RNF43*	2	2	2	9978	9292	51	4.9e-08	1	3.9e-29	2e-14	1	0	0
*ATRX*	1	2	1	11646	12114	60	2.2e-16	1	1e-15	1e-15	1	0	0
*ZBTB20*	2	2	2	8415	6667	43	1.1e-10	0.94	8.6e-19	2.1e-09	1	0	0
*NOTCH1*	1	2	1	10225	7849	55	1e-15	1	1e-15	1e-15	1	0	0
*CDH1*	2	2	2	8634	8254	45	3.6e-16	1	1.1e-23	3.5e-26	1	0	0
*KEAP1*	1	1	1	10060	10030	50	0.4	1	1e-15	0.017	1	1.25e-12	0
*SMARCA4*	1	1	1	6118	6234	30	0.86	1	1e-15	0.93	1	1.11e-16	3.56e-11
*KIT*	1	1	1	15035	10164	74	0.87	1	4.4e-16	0.029	0.95	2.54e-09	1.07e-06
*KMT2B*	2	2	2	8066	6649	44	1.7e-24	1	7e-29	2.3e-24	1	0	0
*NSD1*	1	1	1	13052	8233	67	1.2e-10	0.55	1.9e-14	3.1e-10	1	NA	NA

|DM|: number of differentially methylated genes averaged across *T*_*i*_ ∩ *E*_*i*_ tumor types;

|DE|: number of differentially expressed genes averaged across *T*_*i*_ ∩ *E*_*i*_ tumor types;

$\frac{\mid DM\cap DE\mid }{\mid DE\mid }$ (%): percent of differentially methylated target genes out of differentially expressed target genes, averaged across tumor types *T*_*i*_ ∩ *E*_*i*_

*p*(*DM* ∙ *DE*): p-value testing if number of target genes that are differentially methylated and expression is larger than expected using a hypergeometric distribution combined across tumor types *T*_*i*_ ∩ *E*_*i*_using the Fisher’s method.

*p*(−−), *p*(+−), *p*(−+), *p*(++): p-values that test if number of target genes with “--”,“+-”,“-+”,“++” pattern of methylation and expression changes is larger than expected a using hypergeometric distribution combined across tumor types using the Fisher’s method.

p.methyl: median p-value from testing if the number of overlapping target genes that are differentially methylated by the mutation of the CDG between any pair of two tumor types is larger than expected using a hypergeometric distribution.

p.exp: median p-value from testing if the number of overlapping target genes that are differentially expressed by the mutation of the CDG between any pair of two tumor types is larger than expected using a hypergeometric distribution.

It is clear that across the 26 overlapping driver genes, target genes’ hyper-methylation are significantly associated with their down-regulation (“+-” pattern) and target genes’ hypo-methylation are significantly associated with their up-regulation (“-+” pattern). A specific example of a target gene that is hypo-methylated and up-regulated by the mutation of *TP53* is *HSF1* gene. It is hypo-methylated and up-regulated by *TP53* mutations across 9 tumor types. Dysregulation of chromatin regulators induces global change of chromatin architecture, which is highly interconnected with DNA methylation. DNA methylation and histone modification interact with each other to determine the chromatin state as an euchromatic (on) or heterochromatic (off) state, where euchromatic state is associated with hypomethylation and active gene expression and heterochromatic state is associated with hypermethylation and repressed gene expression [24].

We also investigated the consistency of the differential gene expression and DNA methylation patterns across tumor types. For each CDG, for every pair of tumor types in which it is mutated in more than five samples, we tested using a hypergeometric distribution if the number of overlapping target genes that are differentially methylated by the mutation of the CDG is larger than expected. We then reported the median p-values (Table 5) from all pairs of two tumor types and repeated the same analysis for differential expression. Both median p-values for differential expression and methylation are ‘0’ or close to ‘0’ for most CDGs, which indicates that the differential expression or methylation associated with CDGs are consistent across tumor types. Note that *NSD1* gene was only mutated in one tumor type.

**Fig. 4:**
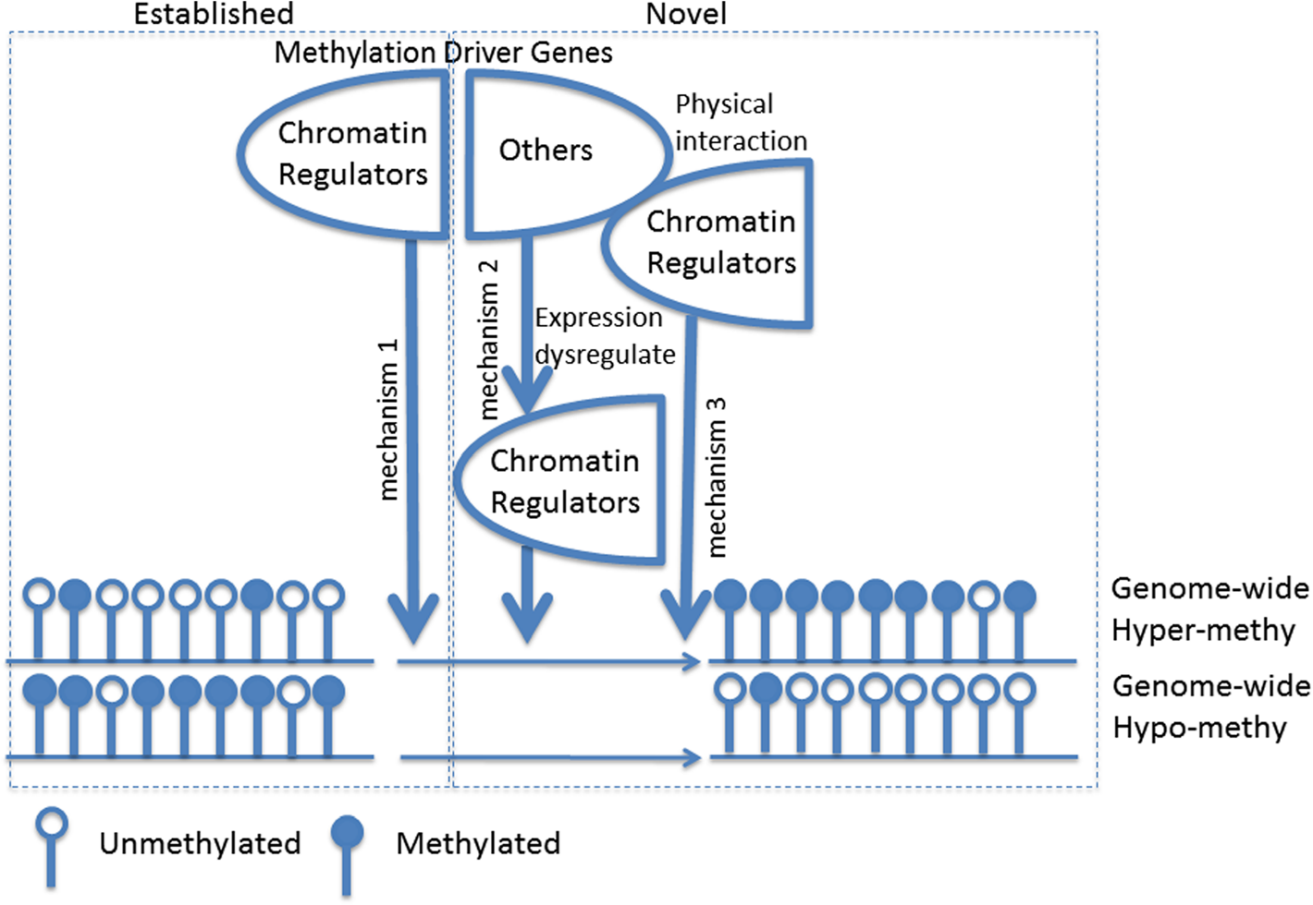
Chromatin remodeling is one of the major mechanisms that amplify the impact of mutations in CDGs by global methylation and gene expression changes

Our findings on how CDG mutations contribute to pan-cancer-associated epigenomic alterations and transcriptomic alterations suggest that there are potentially three mechanisms (Fig. 4): 1) genome-wide methylation and expression changes are associated with changes in chromatin states induced by malfunctions of chromatin regulators directly through mutations of these genes; 2) or indirectly through mutations of other genes that regulate the expression of chromatin regulators; 3) or indirectly through mutations of other genes with which chromatin regulators physically interact with for epigenomic regulation.

## Discussion

We conducted a pan-cancer analysis to identify CDGs whose somatic mutations are associated with genome-wide methylation/expression changes across multiple cancer types. We used a straightforward method to compare methylation/expression levels between mutated and non-mutated groups of each CDG. The MDGs identified highly overlap with the driver genes identified whose mutation states are associated with genome-wide methylation changes by Chen et al. [5] (these overlapping genes are underlined in Table 1), where they used a different method, the Principal Component Analysis (PCA). This provides further validation of the MDGs results. However, our method also identified several MDGs that were not identified by Chen et al. [5] including well-known chromatin regulators *KMT2B*, *KMT2C*, *KMT2D* and *SMARCA4.*

The MDGs that we identified do not overlap the 18 master regulators identified by Yang *et al.* [8]. This is because they did not consider mutation data and focused only on epigenetic enzymes, which exhibit consistently differential expression and DNA methylation instability correlation patterns across cancer types. However, as Table 2 shows, the deregulation of the 18 master regulators in list B are correlated with the mutation status of the MDGs.

Interestingly, the MDGs and EDGs also include genes that are associated with telomere length (TL) elongation in cancers. Telomeres shorten with each cell division, therefore, maintenance of telomere length is critical in tumorigenesis. While telomere shortening is often prevented by activation of telomerase reverse transcriptase (TERT), it is also prevented by a homologous recombination-based process known as alternative lengthening of telomeres (ALT), where remodeling of the telomeric architecture may play a key role [25]. Floris *et al.* [26] recently performed a comprehensive analysis of association between TL and somatic alterations in cancers. To identify TERT-independent TL regulation, they associated somatic alterations of 196 telomere-associated genes to TL ratio between matching tumor and normal samples and found alterations of *ATRX*, *IDH1, TP53, BCOR,* and *RB1* were significantly associated with relative TL elongation under FDR<0.05. Our MDGs include four out of these five genes, suggesting that chromatin remodeling plays an important role in ALT.

In a recent review by Feinberg *et al.* [27], an epigenetic functional classification system was introduced that classifies epigenetic genes into three categories 1) “epigenetic mediators”, which correspond to tumor progenitor genes that are targets of epigenetic modification; 2) “epigenetic modifiers”, which modify DNA methylation or chromatin structure; and 3) “epigenetic modulators”, which influence activities of epigenetic modifiers to destabilize epigenetic states.

Among the 32 MDGs, 8 are well-known chromatin regulators that fall in the category of “epigenetic modifiers”. The remaining 24 genes are considered as new candidates of “epigenetic modulators” that are associated with genome-wide methylation changes through regulating or interacting with chromatin regulators. Further analysis that examined whether mutations of 12 MDGs out of these 24 MDGs are associated with the expression of known epigenetic modifiers across cancer types supports our mechanistic hypothesis that some of these MDGs are the ones that regulate expression of chromatin regulators. Similarly, analysis that examined whether chromatin regulators are enriched among genes that physically interact with the 12 MDGs supports our mechanistic hypothesis that some of these MDGs are the ones that physically interact with chromatin regulators.

Seven out of the 24 MDGs: *PTEN*, *PIK3CA*, *KRAS*, *HRAS*, *BRAF*, *NRAS*, and *KIT* belong to the PI3K/AKT signaling pathway which is known to target and change the function of chromatin-modifying enzymes in SWI/SNF family members [28]. Previous studies provide strong evidence that all these 7 genes are involved in chromatin remodeling. *BRAF* mutation is known to be tightly associated with a CpG island methylator phenotype (CIMP) and alteration of SWI/SNF chromatin remodeling pathway [29]. *RAS* genes which were classified as epigenetic modifiers in the review by Feinberg *et al.* induce global and local chromatin modifications [27]. There is also evidence for direct or indirect interactions between chromatin regulators or chromatin themselves and *KIT* [30], *PIK3CA* [31], and *PTEN* [32].

Another highly enriched function among the identified MDGs is DNA repair. Eight of the 24 MDGs: *TP53*, *PTEN*, *RB1*, *FOXA1*, *BAP1*, *IDH1* and *NF1* are known to play a role in DNA repair, when DNA repair is known to interact with chromatin remodeling. Studies of DNA repair have uncovered that many histone modifications occur after induction of a double-strand break [33]. *TP53* binds to and regulates chromatin regulators, including the methyltransferases *KMT2A* and *KMT2D* and acetyltransferase *KAT6A*, resulting in genome-wide increases of histone methylation and acetylation [34]. *RB1* is also known to bind to and regulate DNA methyltransferase, histone methyltransferases and histone acetyltransferase [35]. *FOXA1* is a pioneer transcription factor whose recruitment to enhancers is associated with DNA demethylation and induction of histone H3 lysine 4 methylation at these enhancers [36, 37]. It was recently uncovered that *FOXA1* interacts with components of DNA repair complexes and that the FOXA1-associated DNA repair complex is implicated in active DNA demethylation [38]. *BAP1*, which is critical for promoting DNA repair by homologous recombination [39], plays a key role in chromatin remodeling by mediating deubiquitination of histone H2A and HCFC1 [40]; *IDH1* is classified as an epigenetic modifier in the review by Feinberg *et al.* [27] and its mutation is known to induce the genome-wide alterations in DNA methylation by inhibiting function of histone and DNA demethylases [41], which also impairs DNA repair [42]. *NF1* is also known to participate in chromatin remodeling activities [43].

For the rest of the MDGs, there is evidence supporting many of their involvement in chromatin modification either by interacting with histone modification enzymes or chromatin remodeling complexes or with chromatin directly, such as *CDH1* [44], *CTNNB1* [45], *EGFR* [46], *KEAP1* [47], *NOTCH1* [48], *STK11*[49], and *ZBTB20* [50]. Especially gene *CIC*, a transcription repressor in the central nervous system identified as the MDG in LGG, physically interacts with a histone methyltransferase *KMT3A* [51]. Note that *CIC* mutations are associated with hyper-methylation in LGG both among *IDH1* wild-type tumors and *IDH1* mutated tumors. Further studies are needed to investigate if the observed clinical and biological impact of *CIC* mutations in LGG is through hyper-methylation of the epigenome.

In this study, we identified CDGs whose somatic mutations are associated with pan-cancer genome-wide methylation/expression changes by using a simple and straightforward method to compare methylation or expression levels between mutated and non-mutated groups of each CDG. We acknowledge that the difference between the two groups may be confounded by other factors, such as mutations in other genes as we observed for *TP53* and *IDH1* in LGG tumors. However, multivariate approaches such as regression models to control for other gene mutations may not be feasible for our purpose due to highly non-normal distribution of methylation levels and sparseness of mutations. Although we focused on associations of somatic mutations with genome-wide methylation and expression changes in this study, this approach can be readily modified to examine association between copy number variations or structural variations with genome-wide methylation and expression changes.

## Conclusions

Our pan-cancer analysis examining connections between somatic mutation and DNA methylation/gene expression identified CDGs (32 MDGs and 29 EDGs) whose somatic mutations are associated with genome-wide methylation/expression changes across multiple cancer types. Many of the identified MDGs are either chromatin regulators or the ones that regulate the expression of or physically interact with chromatin regulators. Twenty-six out of the 29 EDGs overlap with the 32 MDGs. We further confirmed the enrichment of target gene patterns being hyper-methylated and down-regulated or hypo-methylated and up-regulated, by the 26 overlapping genes. These findings highlight that the dysregulation of chromatin regulation is an important mechanism that amplifies the impact of mutations in CDGs by global methylation and gene expression changes.

## Additional files

Additional file 1: **Table S1.** Selection of candidate CDGs across tumor types. (XLSX 9 kb)

Additional file 2: **Table S2.** A. Number of CDGs mutated in at least 5 samples with methylation data in one tumor type; B. Number of CDGs mutated in at least 5 samples with expression data in one tumor type. (XLSX 8 kb)

Additional file 3: **Text S1.** Details with some modified steps for identifying EDGs.; Results of MDGs in LGG; Results from stratified analysis in all other cancers other than LGG. (DOCX 68 kb)

Additional file 4: **Table S3.** Number of genes in the methylation null pool. (XLSX 36 kb)

Additional file 5:**Table S4.** CDGs associated with significant genome-wide methylation changes in one cancer type. Hyper-methylation is defined as CDGs with *pi*,*k* <0.05 and the number of hyper-methylated sites is larger than the number of hypo-methylated sites ($\left|\ {S}_{i,k}^{+}\right|>\left|\ {S}_{i,k}^{-}\right|$). Hypo-methylation is defined as CDGs with *pi*,*k* <0.05 and the number of hyper-methylated sites is smaller than the number of hypo-methylated sites ($\left|\ {S}_{i,k}^{+}\right|\le \left|\ {S}_{i,k}^{-}\right|$). (XLSX 11 kb)

Additional file 6: **Figure S1.** Mutation patterns of the identified 32 MDGs across 20 TCGA tumor types. Each row represents a tumor sample and each column represents a MDG. Light color indicates no mutation and dark color indicates mutations. (TIF 287 kb)

Additional file 7: **Table S5.** Results from stratified analysis for the rest of the 15 cancer types (other than LGG) whose genome-wide methylation patterns were affected by one of the identified 32 major MDGs. (XLSX 10 kb)

Additional file 8: **Table S6.** Number of tumor samples with mutations in each of the 21 chromatin regulator genes in list B. (XLSX 10 kb)

Additional file 9: **Table S7.** Chromatin regulators dysregulated by *IDH1* and *CIC* in LGG. |*Ti*| is number of tumor types whose genome-wide methylation levels are significantly associated with the mutation status of CDG *i*; *N*_*1*_ is number of genome-wide genes whose expression levels are dysregulated by MDG *i* in all |*Ti*| tumor types; *N*_*2*_ is number of genes in list A that are dysregulated in all |*Ti*| tumor types; *N*_*3*_ is number of genes in list B that are dysregulated in all |*Ti*| tumor types; Enrichment Pvalue_A_ and Pvalue_B_ are calculated using hypergeometric distributions testing if genes in lists A and B occur more frequently than expected by random chance among genome-wide differentially expressed target genes. (XLSX 9 kb)

Additional file 10:**Table S8**. CDGs associated with significant genome-wide expression changes in one cancer type. Up-regulation is defined as CDGs with ${p}_{i,k}^{\prime }$<0.05 and the number of up-regulated genes is larger than the number of down-regulated genes ($\left|\ {G}_{i,k}^{+}\right|>\left|\ {G}_{i,k}^{-}\right|$). Down-regulation is defined as CDGs with ${p}_{i,k}^{\prime }$<0.05 and the number of up-regulated genes is smaller than the numsber of down-regulated genes ($\left|\ {G}_{i,k}^{+}\right|\le \left|\ {G}_{i,k}^{-}\right|$). (XLSX 9 kb)

Additional file 11: **Table S9.** Correlation between methylation and expression changes in each cancer type. K_1_ is number of CDGs mutated in ≥ 5 samples with expression data available; K_2_ is number of CDGs mutated in ≥ 5 samples with methylation data available; E is set of CDGs with *p*_*i,k*_’<0.05, that is, CDGs whose mutation status are significantly associated with f differentially expressed genes; M is set of CDGs with *p*_*i,k*_<0.05, that is, CDGs whose mutation status are significantly associated with differential methylation; ‘cor’ stands for correlation between the number of differentially methylated sites and the number of differentially expressed genes by the CDG mutation. (XLSX 9 kb)

Additional file 12: **Figure S2.** Significance of overlap between genome-wide up/down-regulation and hyper/hypo-methylation associated with the mutation status of the overlapping driver genes. (a) We examined signature patterns of target genes’ promotor regions methylation and expression changes by the overlapping driver genes, *i.e.*, target genes that are hyper-methylated and up-regulated by overlapping driver gene *i*, the “++” pattern; target genes that are hyper- methylated and down-regulated by overlapping driver gene *i*, the “+-” pattern; target genes that are hypo-methylated and up-regulated by overlapping driver gene *i*, the “-+” pattern; and target genes that are hypo-methylated and down- regulated by overlapping driver gene *i*, the “–” pattern. (b) We calculated a p-value that tests if number of target genes that are differentially methylated and expressed is larger than expected using a hypergeometric distribution, and a p-value that tests if number of target genes with one of the 4 pattern of methylation and expression changes is larger than expected using a hypergeometric distribution, where we combined per tumor type p-values across tumor types using the Fisher’s method. (TIF 94 kb)

## Abbreviations

BLCA: Bladder urothelial carcinoma
BRCA: Breast invasive carcinoma
CDG: Cancer driver genes
CESC: Cervical squamous cell carcinoma
COAD: Colon adenocarcinoma
EDG: Expression driver gene
GBM: Glioblastoma
HNSC: Head and neck squamous cell carcinoma
KIRC: Kidney renal clear cell carcinoma
KIRP: Kidney renal papillary cell carcinoma
LGG: Acute myeloid leukemia (LAML), lower grade glioma
LIHC: Liver hepatocellular carcinoma
LUAD: Lung adenocarcinoma
LUSC: Lung squamous cell carcinoma
MDG: Methylation driver gene
PAAD: Pancreatic adenocarcinoma
PCPG: Pheochromocytoma and paraganglioma
PRAD: Prostate adenocarcinoma
SARC: Sarcoma
STAD: Stomach adenocarcinoma
TCGA: The Cancer Genome Atlas
TERT: Telomerase reverse transcriptase
THCA: Thyroid carcinoma
TL: Telomere length
UCEC: Uterine corpus endometrial carcinoma
