# Supplementary material for: A pan-cancer analysis of driver gene mutations, DNA methylation and gene expressions reveals that chromatin remodeling is a major mechanism inducing global changes in cancer epigenomes
Source: BMC Med Genomics. 2018 Nov 6;11:98. doi: 10.1186/s12920-018-0425-z (PMC6218985; doi:10.1186/s12920-018-0425-z)
Supplement: Supplementary file 3 — Text S1. Details with some modified steps for identifying EDGs.; Results of MDGs in LGG; Results from stratified analysis in all other cancers other than LGG. (DOCX 68 kb) [file 12920_2018_425_MOESM3_ESM.docx]

**Supplementary material to**

**A pan-cancer analysis of driver gene mutations, DNA methylation and gene expressions reveals that chromatin remodeling is a major mechanism inducing global changes in cancer epigenomes.**

**Ahrim Youn, Kyung In Kim, Raul Rabadan, Benjamin Tycko, Yufeng Shen, Shuang Wang**

**1. Pan-cancer analysis to identify EDGs**

**Associate CDGs and gene expression in one cancer type**

For CDG *i*, we used t-test instead of Wilcoxn test since for RNA-Seq data, the deviation from normality is not that severe. We define a set of genome-wide up-regulated genes, $G_{i,k}^{+}$ whose expression levels are significantly (with each having two-sample t-test one-sided p-values < 0.05) increased in the mutated group comparing to the non-mutated group of driver gene *i* in cancer type *k*. Similarly, we define a set of down-regulated genes $G_{i,k}^{-}$, whose expression levels are significantly decreased in the mutated group. Thus we define a set of genes $G_{i,k}= G_{i,k}^{+}\cup G_{i,k}^{-}$ whose expression levels are either up- or down-regulated in samples where CDG *i* was mutated in enough samples of cancer type *k*. We use a larger cutoff for expression data since there are fewer genes (20K) than CpG sites (450K).

We then test if mutation status of CDG *i* is associated with genome-wide up- or down-regulated changes in one cancer type. Here we use the “expression null pool” generated in the same way as the “methylation null pool”. The only difference is that for the “expression null pool”, we match mutation data with expression data while for the “methylation null pool”, mutation data are matched with methylation data. We ended up with 6,793 genes across 20 tumor types in the “expression null pool”. We calculate the p-value $p_{i,k}^{'}$ to test if driver gene *i* is significantly associated with genome-wide up- or down-regulated changes in cancer type *k* using the “expression null pool” as the null distribution to compare with, $p_{i,k}^{'}=\frac{1}{6793}\sum_{j_{null}=1}^{6793} I(n_{i,k}^{e}\leq n_{j_{null}}^{e} ).$

We also classify the effect of CDG *i* on genome-wide expression in tumor type *k* as:

**Associate CDGs and gene expression across multiple cancer types**

We similarly use a resampling method from the “expression null pool” to calculate the p-value,$p_{i}^{'}$ that tests if CDG *i* mutation is associated with genome-wide expression changes across multiple cancer types. To calculate $p_{i}^{'}$, we compare the observed total number of differentially expressed genes associated with the mutation status of CDG *i* summed over $A_{i}^{'}$ cancer types, , to *B* resampled values generated from the “expression null pool” where we set *B*=one million. More specifically, for CDG *i* that is mutated in$|A_{i}^{'}|$ number of tumor types, the null distribution is generated using the *B* sets of sum of $|A_{i}^{'}|$ random samples from the “expression null pool”. We can calculate $p_{i}^{'}$ as follows:

$p_{i}^{'}=\sum_{b=1}^{B} I(\sum_{k\in A_{i}^{'}} n_{i,k}^{e}\leq\sum_{j=1}^{|A_{i}^{'}|} n_{r_{b,j}}^{e})/B$ ,

where$\sum_{j=1}^{|A_{i}^{'}|} n_{r_{b,j}}^{e}$ is sum of $|A_{i}^{'}|$random samples from the “expression null pool” with $n_{j_{null}}^{e}$, $j_{null}=1,\ldots,6793$ and $r_{b,j}$ is random number between 1 and 6793 for the b^th^ resampling. We use Benjamini-Hochberg procedure to adjust for multiple comparisons on $p_{i}^{'}.$ The adjustment is done within the group of driver genes that were mutated in the same number of tumor types.

**2. MDGs in LGG**

In LGG, *TP53*, *IDH1*, *CIC*, *ATRX*, and *NOTCH1* are identified as the MDGs that are associated with more hyper-methylated CpG sites genome-wide, while *PTEN*, *RB1*, *NF1*, and *EGFR* are identified as the MDGs that are associated with more hypo-methylated CpG sites genome-wide (Table 1). Similarly as we observed that *TP53* and *IDH1* mutations co-occur together, almost all LGG tumors with mutations at *CIC*, *ATRX*, or *NOTCH1* (that are associated with hyper-methylation) also have *IDH1* mutations, while almost all LGG tumors with mutations at *PTEN*, *RB1*, *NF1*, or *EGFR* (that are associated with hypo-methylation) have wild-type *IDH1* (Figure 2). It is known that tumors with wild-type *IDH1* were molecularly and clinically distinct from subtypes with mutated *IDH1* (1).

Given the prominent role of *IDH1* in LGG, we stratified LGG tumor samples by the *IDH1* mutation status and further examined the effect of the other 31 MDGs within the *IDH1* mutation stratum and the *IDH1*wild-type stratum. Within the *IDH1* mutation stratum, *CIC* and *NOTCH1* mutations remain to be significantly associated with more CpG sites to be hyper-methylated genome-wide, while *ATRX* and *TP53* mutations are changed to be significantly associated with more hypo-methylation genome-wide. Within the *IDH1* wild-type stratum, *CIC* mutations remain to be significantly associated with hyper-methylation genome-wide, while *TP53* mutations are changed to be associated with more hypo-methylated CpG sites genome-wide with marginal significance. Thus *CIC* is indeed associated with hyper-methylation in LGG independent of *IDH1* mutation status. Although *CIC* gene is frequently mutated in LGG tumors, its clinical and biological impact is not very well understood. A recent study suggested that *CIC* mutations result in activation of proliferative pathways, and inactivation of *CIC* gene then leads to an upregulation of genes involved in cell proliferation (2). Further studies are needed to investigate if the observed clinical and biological impact of *CIC* mutations is through hyper-methylating the epigenome.

**3. Further Stratified Analysis in all other cancers reveals similar conclusions**

The stratified analysis in LGG confirmed that *TP53* mutations are consistently associated with genome-wide hypo-methylation across all cancer types in which it significantly affects genome-wide methylation patterns. We performed similar stratified analysis in all other tumor types whose genome-wide methylation patterns were significantly associated with mutations of the identified MDGs and observed similar patterns as in the non-stratified analysis (Supplementary Table S4). Specifically, for each tumor type, we first identified the MDG whose mutation status is associated with the largest number of CpG sites to be differentially methylated (e.g., in LGG, *IDH1* mutation is associated with significant methylation changes in the largest number of CpG sites). We then stratified tumor samples by the mutation status of this MDG into mutated stratum and wild-type stratum. For the rest of the 31 MDGs mutated in the tumor type studied, we tested if their mutations are associated with significant methylation changes genome-wide within each of the two strata and also determined whether they are hyper- or hypo- MDGs. That is, within the mutated stratum and within the wild-type stratum, whether the mutation status of these MDGs hyper- or hypo-methylate more CpG sites genome-wide.
