# Supplementary figures and images for: A pan-cancer analysis of driver gene mutations, DNA methylation and gene expressions reveals that chromatin remodeling is a major mechanism inducing global changes in cancer epigenomes
Source: BMC Med Genomics. 2018 Nov 6;11:98. doi: 10.1186/s12920-018-0425-z (PMC6218985; doi:10.1186/s12920-018-0425-z)

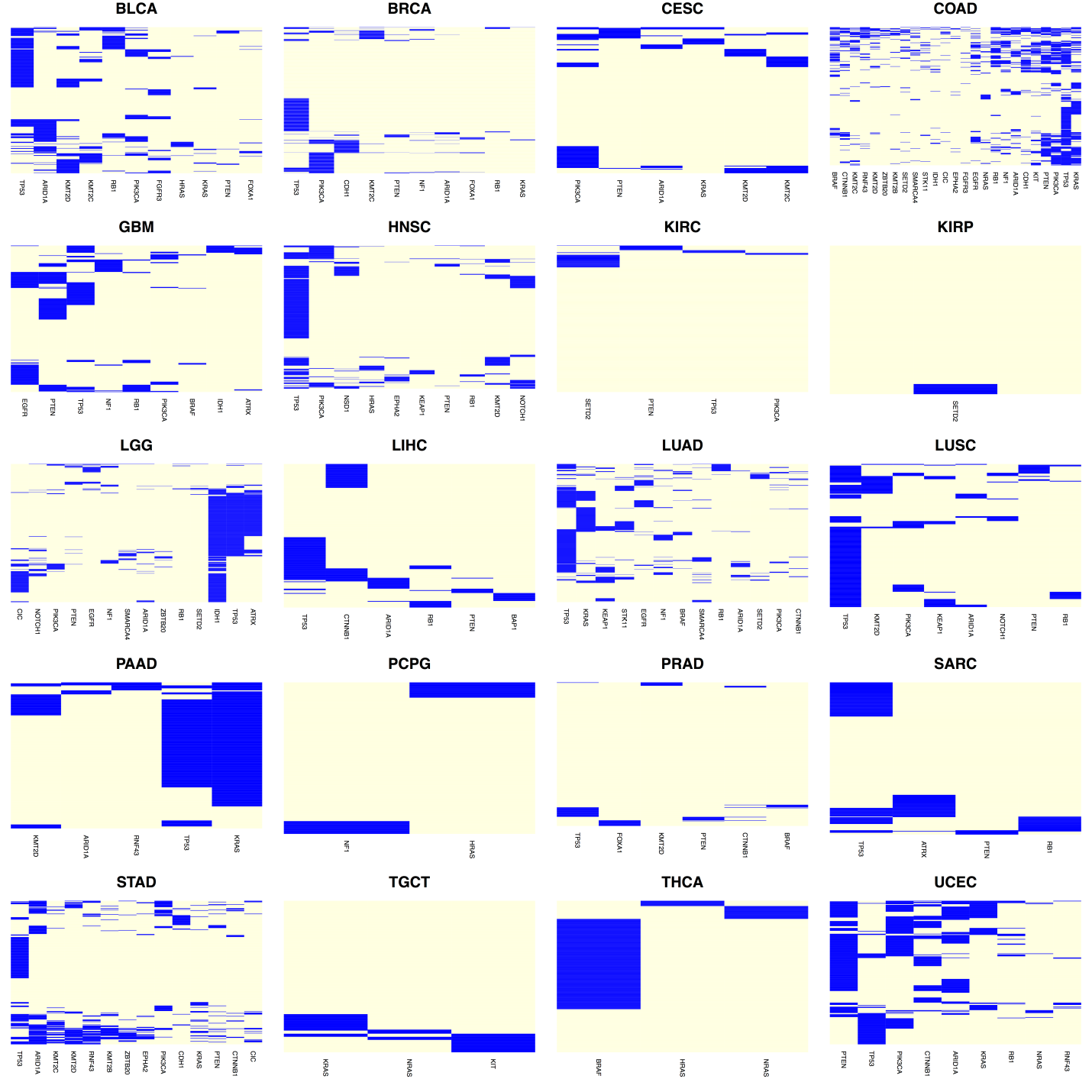

Supplement: Supplementary file 6 — Figure S1. Mutation patterns of the identified 32 MDGs across 20 TCGA tumor types. Each row represents a tumor sample and each column represents a MDG. Light color indicates no mutation and dark color indicates mutations. (TIF 287 kb) [file 12920_2018_425_MOESM6_ESM.tif]

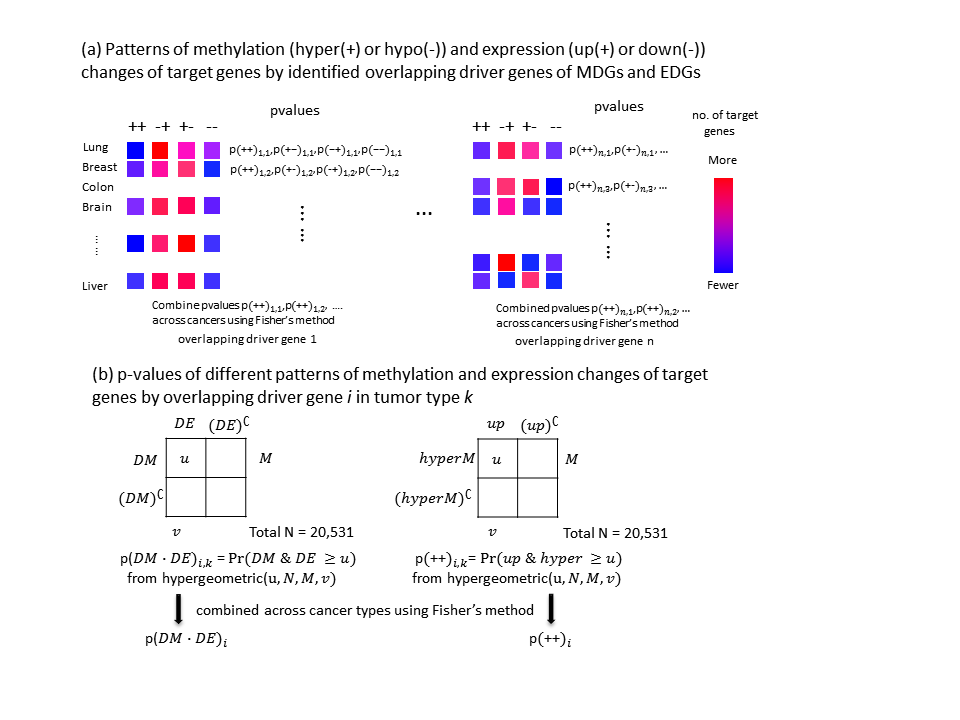

Supplement: Supplementary file 12 — Figure S2. Significance of overlap between genome-wide up/down-regulation and hyper/hypo-methylation associated with the mutation status of the overlapping driver genes. (a) We examined signature patterns of target genes’ promotor regions methylation and expression changes by the overlapping driver genes, i.e., target genes that are hyper-methylated and up-regulated by overlapping driver gene i, the “++” pattern; target genes that are hyper- methylated and down-regulated by overlapping driver gene i, the “+-” pattern; target genes that are hypo-methylated and up-regulated by overlapping driver gene i, the “-+” pattern; and target genes that are hypo-methylated and down- regulated by overlapping driver gene i, the “–” pattern. (b) We calculated a p-value that tests if number of target genes that are differentially methylated and expressed is larger than expected using a hypergeometric distribution, and a p-value that tests if number of target genes with one of the 4 pattern of methylation and expression changes is larger than expected using a hypergeometric distribution, where we combined per tumor type p-values across tumor types using the Fisher’s method. (TIF 94 kb) [file 12920_2018_425_MOESM12_ESM.tif]
